# Supplementary material for: Arginine Regulates Skeletal Muscle Fiber Type Formation via mTOR Signaling Pathway
Source: Int J Mol Sci. 2024 Jun 4;25(11):6184. doi: 10.3390/ijms25116184 (PMC11173221; doi:10.3390/ijms25116184)
Supplement: Supplementary file 1 [file ijms-25-06184-s001.zip › ijms-3021649-supplementary.pdf]

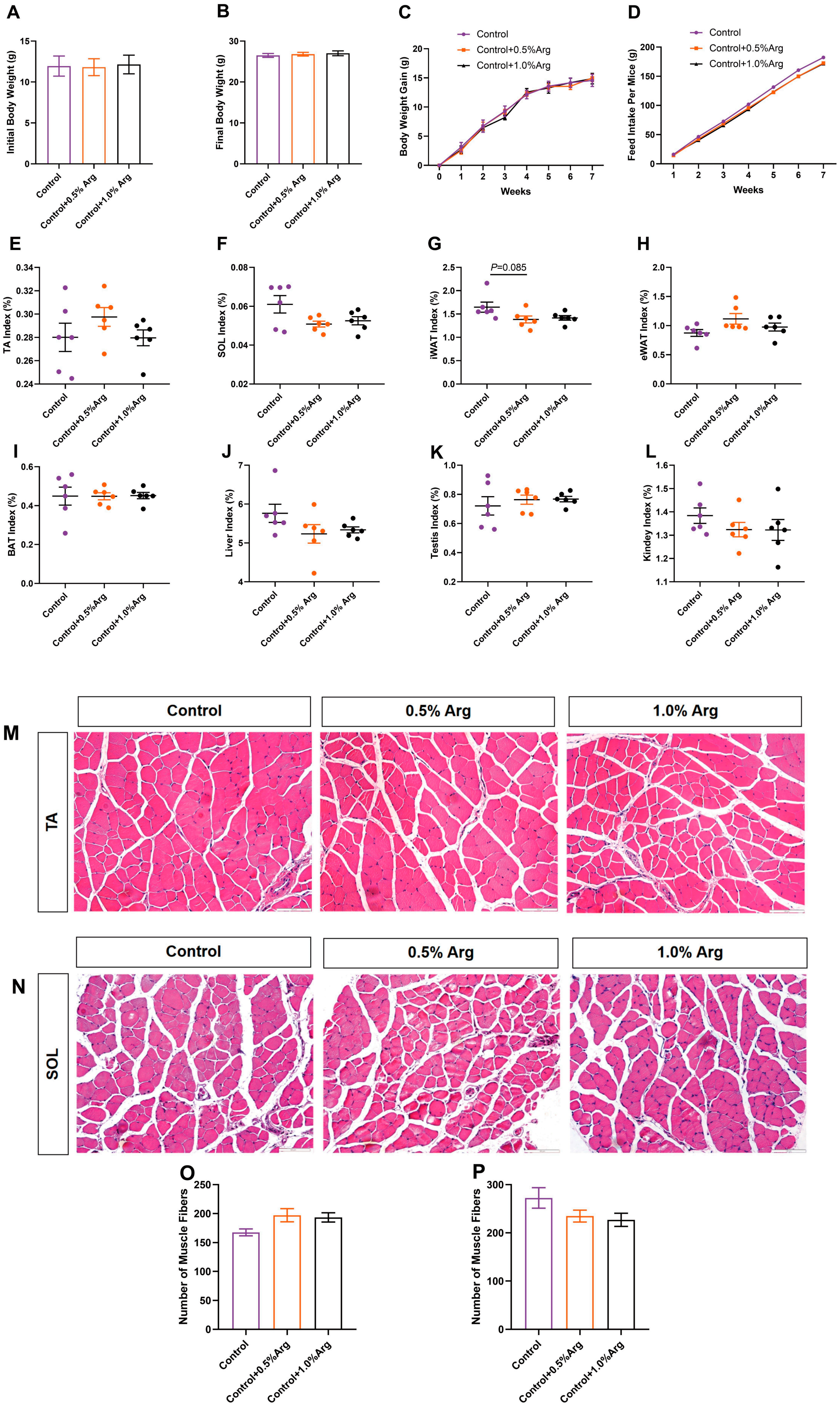

A
Initial Body Weight (g)
Control
Control+0.5% Arg
Control+1.0% Arg
B
Final Body Wight (g)
C
Control
Control+0.5%Arg
Control+1.0%Arg
Body Weight Gain (g)
Weeks
D
Feed Intake Per Mice (g)
Weeks
E
TA Index (%)
F
SOL Index (%)
G
P=0.085
iWAT Index (%)
H
eWAT Index (%)
I
BAT Index (%)
J
Liver Index (%)
K
Testis Index (%)
L
Kindey Index (%)
M
TA
Control
0.5% Arg
1.0% Arg
N
SOL
O
Number of Muscle Fibers
P
Number of Muscle Fibers

Figure S1: Effects of Arg on growth performance and organ index in mice. (A-D) Initial body weight (A), final body weight (B), body weight gain (C), feed intake per mice (D). (E-L) Tibialis anterior muscle index (E), soleus muscle index (F), inguinal white adipose tissue index (G), epidinymal white adipose tissue index (H), brown adipose tissue index (I), liver index (J), testis index (K), kindey index (L). (M-P) Representative images of HE staining of tibialis anterior (M) and soleus (N) muscles, number of muscle fiber in fixed area was based on HE staining in tibialis anterior (O) and soleus (P) muscles. Scale bar in (M) and (N) represent 100 μm.
